# Ceramic Ti—B Composites Synthesized by Combustion Followed by High-Temperature Deformation

**DOI:** 10.3390/ma9121027

**Published:** 2016-12-20

**Authors:** Pavel M. Bazhin, Alexander M. Stolin, Alexander S. Konstantinov, Elena V. Kostitsyna, Andrey S. Ignatov

**Affiliations:** 1 Institute of Structural Macrokinetics and Materials Sciences, Russian Academy of Sciences, ul. Akademika Osipyana 8, Chernogolovka 142432, Russia; olimp@ism.ac.ru (P.M.B.); amstolin@ism.ac.ru (A.M.S.); slaity@mail.ru (A.S.K.); 2 Department of Functional Nanosystems and High-Temperature Materials, National University of Science and Technology MISiS, Leninskii pr. 4, Moscow 119049, Russia; ignatov@misis.ru

**Keywords:** ceramic composite material, combustion, high-temperature deformation, self-propagating high-temperature synthesis, SHS extrusion

## Abstract

Long compact cylindrical rods, which consist of a titanium monoboride-based TiB—30 wt % Ti ceramic composite material, are synthesized during combustion of the initial components (titanium, boron) followed by high-temperature deformation. High-temperature deformation is found to affect the orientation of the hardening titanium monoboride phase in the sample volume and the phase composition of the sample. The combustion temperature is studied as a function of the relative density of the initial workpiece under the experimental conditions.

## 1. Introduction

The refractory oxygen-free compounds of elements with boron, namely, borides, are widely used in engineering. These compounds have metallic properties and their electrical conductivity is very high. They are characterized by a high refractory property (2500–3500 °C), hardness (sometimes as high as the hardness of diamond), wear resistance, and chemical resistance to aggressive media. However, they are brittle. Boride coatings strongly increase the hardness, the chemical resistance, and the wear resistance of the products made of them.

One of the fields of boride application is the use of borides as a matrix material for metal-matrix composites [1,2,3]. Such composites combine the plasticity and the fracture toughness of a titanium matrix with a high strength, hardness, rigidity, and heat resistance. The fraction of ceramic alloys and composites based on titanium borides is constantly growing in rocket production, aviation engineering, and car industry, and they also have applications as biological implants and hard high-temperature coatings in various fields of engineering.

The main methods of producing compact products from these materials for various functional purposes are casting and powder metallurgy [4,5,6]. Spark plasma sintering is being rapidly developed to produce ceramic compacted materials with minimum porosity [7,8,9]. However, these methods need much energy and time and include a large number of technological stages.

An alternative to these methods is one of the self-propagating high-temperature synthesis (SHS) methods [10,11], namely, SHS extrusion. This method combines combustion and high-temperature deformation of the materials to be synthesized and can be used to form compact products as long as 400–500 mm with a residual porosity of at most 5% at one technological stage in several tens of seconds [12,13,14]. Note that these high-density rods made of titanium boride can be used as valves in the gas-distribution system of a modern car, which are now produced by powder metallurgy.

The purpose of this work is to synthesize cylindrical rods 3 and 4 mm in diameter and 300 mm in length from a TiB—30 wt % Ti ceramic material.

The microstructure, the microhardness, and the porosity of the synthesized materials are studied.

The synthesized materials are subjected to heat-resistance tests in an oxidizing atmosphere at a temperature of 900 and 1100 °C.

## 2. Materials and Methods

We studied a ceramic TiB—30 wt % Ti composite material. Commercial titanium (PTS grade, 81.6 wt %) and boron (amorphous black boron, 18.4 wt %) powders were taken in the proportion that provides the formation of titanium monoboride. Excess titanium (30 wt %) was introduced into the initial mixture to achieve the best high-temperature deformability [14].

The preliminarily mixed charge was compressed into cylindrical 35-g rods, with a diameter of 25 mm and a relative density of 0.52–0.58 (Table 1). A combustion SHS reaction was initiated, and the synthesized material was then subjected to high-temperature deformation (SHS extrusion [12,13,14]) through a die 3 or 4 mm in diameter at a strain of 0.99 or 0.97, respectively, 3–5 s after the reaction. The press plunger speed was 60 mm/s and the pressing pressure was 50 MPa.

The combustion temperature of selected compounds was measured using tungsten-rhenium thermocouples. Based on the thermograms obtained, the rate of combustion was calculated by means of the formula [15]:

*U* = *l*/*t* (1 + ε) (1)

Here, U is the actual rate of combustion, whereas *U_0_* = *l*/*t* is the measured rate of combustion. The latter is calculated as the distance between thermocouples (*l*, mm) divided by the time necessary for the combustion’s wave propagation between them (*t*, c). Next, ε = (*L* − *L*_0_)/*L*_0_ is the relative extension of the sample during combustion, where *L*_0_ is the initial length and L is the length of the sample after combustion.

This formula takes into account the important condition of the synthesis, i.e., the increase in the sample length during the process, so that the measured combustion rate turns out to be less than the true rate of combustion. According to this formula, the difference between the actual and measured combustion rates is determined by the value of sample macrode formation, related to the change of the initial sample length.

The laboratory heat-resistance tests of the synthesized materials were performed according to State Standard GOST 21910-76, and three cylindrical rods of each composition that were 3 mm in diameter and 30 mm in length were tested. The samples of the same composition have been obtained by SHS-extrusion at different experimental parameters. This was done to establish the identity of properties of the samples obtained in different experiments. The heat resistance was determined by weighing samples 3 mm in diameter on a Vibra HT analytical balance (step of 10^−4^ g, accuracy class “special 1”) when they were held in a furnace in an oxidizing air atmosphere at a temperature of 900 °C for 2, 4, 9, and 16 h. Samples of 4 mm in diameter were tested after holding in a furnace in an oxidizing air atmosphere at 1100 °C for 1, 3, 5, 7, and 10 h.

X-ray diffraction (XRD) analysis was performed on a DRON-3 diffractometer. A microstructure was examined with an ultrahigh-resolution Ultra plus (Carl Zeiss, Jena, Germany) field emission scanning electron microscope (SEM). The porosity was measured by hydrostatic weighing, the microhardness was determined using a PMT-3 microhardness tester.

The actual speed of the penetration corrosion (*V_h_*) is the time derivative of the uniform corrosion depth as a function of time. It was calculated as follows:

(2) $$V_{h}=\frac{d_{h}}{d_{t}}=\frac{1}{\rho }\times \frac{d_{q}}{d_{t}}=\frac{1}{\rho \cdot S}\times \frac{d_{m}}{d_{t}}$$

Here, *ρ* is the density, and *d_h_* is the thickness of the oxidized layer.

## 3. Results and Discussion

The ceramic material was synthesized according to the mechanism of reaction diffusion: titanium in the combustion zone melts and interacts with boron to form titanium monoboride. Since titanium was taken in excess, it also played the role of a matrix in the material to be synthesized and increased the plasticity during high-temperature deformation.

**Figure 1:**
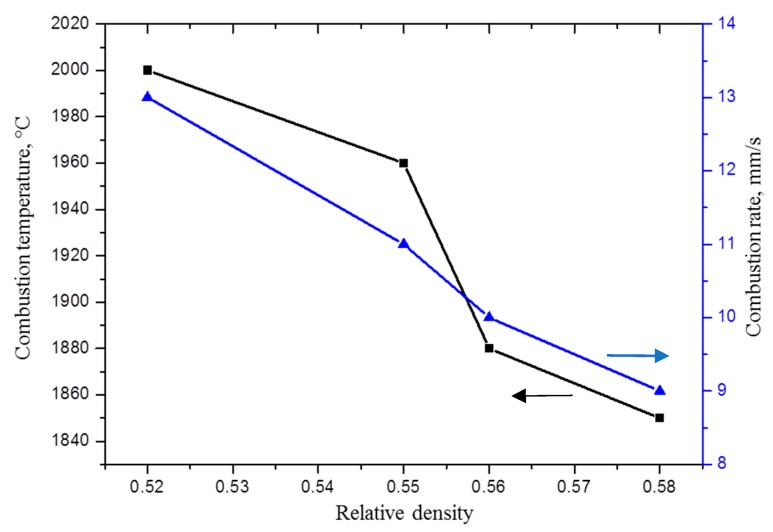
Temperature and rate of combustion vs. the relative density of a charge sample.

The adiabatic combustion temperature of the system was 2200 °C. Under the real high-temperature deformation conditions, the combustion temperature decreases from 2000 to 1800 °C as the relative density of the charge sample increases from 0.52 to 0.58. (Figure 1).

This behavior can be explained by the fact that, as the relative density of the charge sample increases, the contact between the initial reagents increases, the heat conduction from the combustion products to the initial components and the mold walls increases, and the combustion temperature decreases. We failed to form an intact initial workpiece with a relative density lower than 0.52. Based on our experiments, we found that initial charge workpieces for SHS extrusion should have a relative density *ρ_rel_* = 0.52, and the combustion rate under these conditions was 13 mm/s. These parameters ensure the maximum combustion temperature, which increases the time interval within which the material to be synthesized has high-temperature deformability. As a result, the process of SHS extrusion was highly reproducible and the quality of extruded rods was improved [12].

**Figure 2:**
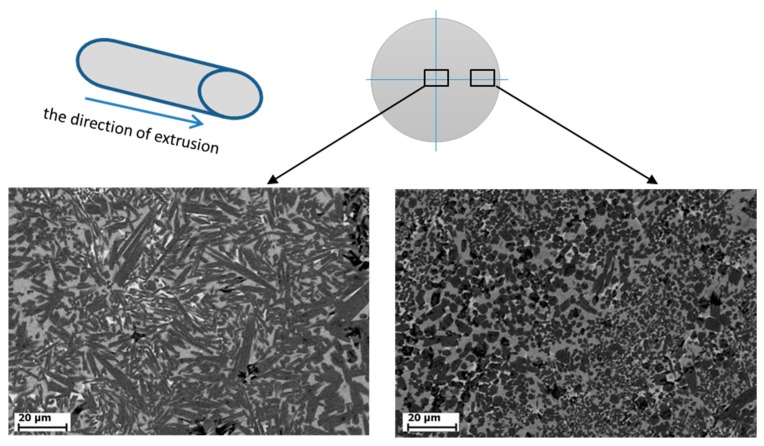
Microstructure of the cross section of the material synthesized during combustion followed by high-temperature deformation as follows from EDS (energy-dispersive X-ray spectroscopy) data.

The microstructure of the cross section of the extruded material is similar at various strains and consists of titanium boride particles (Figure 2, dark gray regions) located in a titanium matrix (Figure 2, light gray region). Based on the reported data [16], we found that the (010) predominant growth of a titanium monoboride crystal causes the formation of TiB in the form of fibers or whiskers, the length of which is much larger than the transverse sizes. In the cross section, the fibers form hexagons faceted by planes (100), (101), and (101^−^). The TiB grains at the sample surface in the microstructure of the synthesized materials have a predominant longitudinal orientation because of high stresses directed along a material flow and friction on the die walls. These grains have an irregular polygonal shape in the cross section and sizes from 1–2 to 5–6 µm. Lower stresses are operative in the central part of the sample, and TiB fibers are oriented along a material flow to a lesser extent and are directed along two directions. Grains at the center coarsen due to slower heat removal in cooling as compared to the sample surface. The grain sizes in the cross section are up to 8–10 µm, the fiber thickness in the (010) longitudinal direction is from 1 to 2–5 µm, and the fiber length is from 2–3 to 40–45 µm.

**Figure 3:**
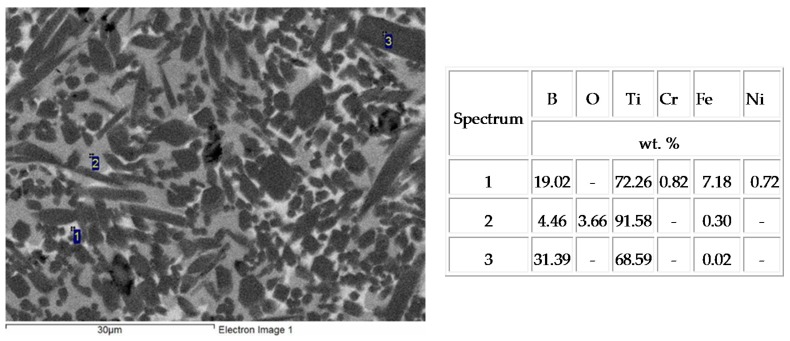
Microstructure and the results of energy dispersive analysis of elements in the material synthesized during combustion followed by high-temperature deformation.

**Figure 4:**
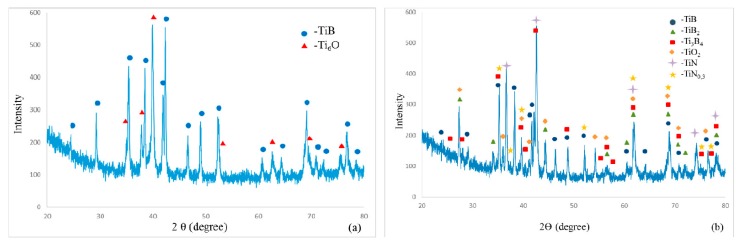
Results of X-ray diffraction (XRD) analysis of the ceramic materials synthesized by combustion during (**a**) self-propagating high-temperature synthesis (SHS) followed by high-temperature deformation (SHS extrusion) and (**b**) SHS.

Iron, chromium, and nickel impurities in the initial components are detected over the polished section area (Figure 3, spectrum 1); as follows from EDS (energy-dispersive X-ray spectroscopy) data, their content is at most 5 wt %. The results of energy dispersive analysis and XRD demonstrate that oxygen dissolves in molten titanium to form the Ti_6_O solid solution during synthesis and subsequent high-temperature deformation (Figure 4a). Energy dispersive analysis shows that the mass fraction of oxygen in the solid solution is at most 4%–5% (Figure 3, spectrum 2). For comparison, we synthesized a similar material without high-temperature deformation in air. The synthesized material consisted of six phases, and the main phases were titanium monoboride TiB, titanium nitrides TiN and TiN_0.3_, titanium oxide TiO_2_, and Ti_3_B_4_ (Figure 4b). The main fraction of titanium oxides and nitrides concentrated at the material surface. The initial mixture composition was calculated to form the end material TiB—30% Ti; however, excess boron appeared due to the high chemical activity of titanium and its interaction with the air components. As a result, additional phases TiB_2_ and Ti_3_B_4_ appeared. Although contact of air with the reaction zone is hindered during SHS extrusion, oxygen dissolves partly in titanium to form the Ti_6_O solid solution during synthesis and subsequent high-temperature deformation. Note that the material synthesized by SHS without deformation had a porosity of 45%–50%, and the distribution of the structural constituents over the volume was non-uniform. Combustion followed by high-temperature deformation leads to a uniform distribution of the structural constituents and to a decrease in the porosity to 2%–4%.

**Figure 5:**
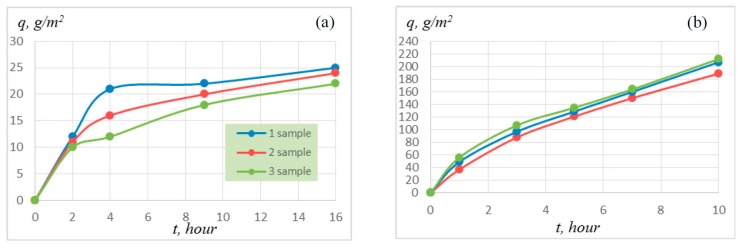
Specific sample mass increment at (**a**) 900 °C and (**b**) 1100 °C.

**Figure 6:**
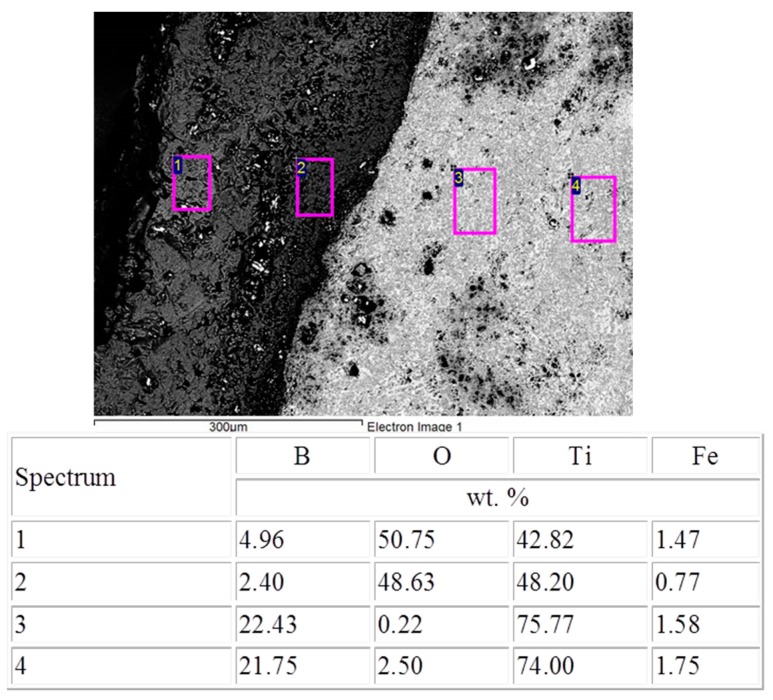
SEM images of the sample after oxidation at 1100 °C for 10 h.

The synthesized materials were oxidized at a temperature of 900 and 1100 °C. In the first oxidation hours, the sample mass increment is maximal because of intense oxidation of the material surface and the formation of oxide films, which hinder the penetration of oxygen into the material volume (Figure 5). The oxide layer thickness on the sample surface was found to be 80–120 µm after holding in a furnace at 900 °C for 15 h, and this thickness was 170–200 µm (Figure 6) after holding at 1100 °C for 10 h.

**Figure 7:**
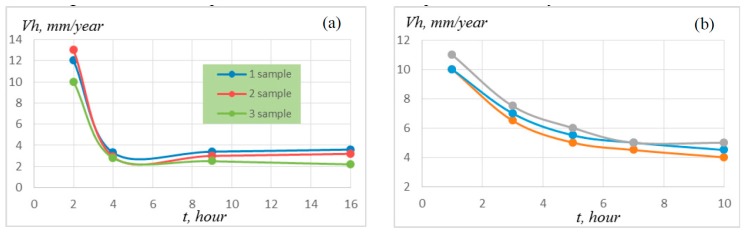
Average rate of corrosion penetration in an oxidized sample at (**a**) 900 °C and (**b**) 1100 °C.

The average rate of corrosion penetration in an oxidized sample was 10–13 mm/year in the first oxidation hours, and this rate decreased to 2–4 mm/year after holding for 5 h at an oxidation temperature of 900 °C (Figure 7). At an oxidation temperature of 1100 °C, this dependence leveled off after oxidation for 6–7 h, and the average rate of corrosion penetration in an oxidized sample was 4–5 mm/year.

**Figure 8:**
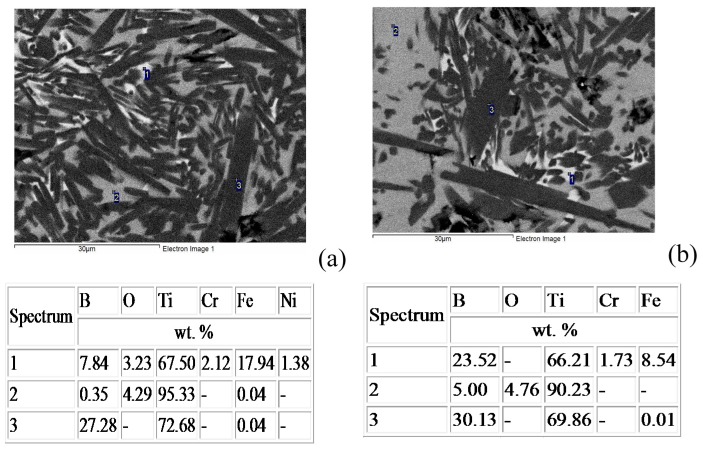
Microstructure and the results of energy dispersive analysis of elements after oxidation at 1100 °C: (**a**) central part and (**b**) sample surface.

After oxidation, the material exhibited no structural and chemical changes, the TiB grain sizes did not grow in both directions, and no cracks and pores were detected in the material volume (Figure 8). The hardness measured at a load of 100 g before and after oxidation was 12–13 GPa and 11–12 GPa, respectively. Based on these data, we can state that the synthesized materials are heat resistant up to a temperature of 1100 °C.

## 4. Conclusions

Titanium monoboride-based ceramic materials with a porosity of 2%–4% were synthesized during SHS combustion followed by high-temperature deformation. These materials were found to consist of the hardening TiB phase and a titanium matrix with 5 wt % oxygen incorporated in the crystal lattice.

Under high-temperature deformation conditions, the combustion temperature of the TiB—30 wt % Ti material was 1800–2000 °C, and this temperature decreased when the relative density of a charge workpiece increased from 0.55 to 0.58.

The deformation conditions influence the orientation of the hardening titanium monoboride phase. The TiB fibers at the sample surface have a predominant longitudinal orientation because of high stresses directed along a material flow and friction on the die walls. Lower stresses are operative in the central part of the sample, and TiB fibers are oriented along a material flow to a lesser extent and are directed along two directions.

Heat-resistance tests of the synthesized materials showed that the specific sample mass increment was 22–25 g/m^2^ at a temperature of 900 °C for 15 h and was 190–212 g/m^2^ at 1100 °C for 15 h. The average rate of corrosion penetration in an oxidized sample did not exceed 4–5 mm/year.

Upon heating to 1100 °C, the material exhibited no structural and chemical changes, the grain sizes of the hardening titanium monoboride phase did not grow, and no cracks and pores were detected in the material volume.

## Figures and Tables

**Table 1:** Characteristics of the initial samples.

Height Green Sample, mm	Density Green Sample, g/cm^3^	Theoretical Density Compact Green Sample, g/cm^3^	Relative Density
32	2.23	3.85	0.58
33	2.16		0.56
34	2.1		0.55
35	2.0		0.52
